# Establishment of an Indirect ELISA for Bovine Infectious Rhinotracheitis Based on Eukaryotic Expression of gD Protein in CHO Cells

**DOI:** 10.3390/vetsci13070699

**Published:** 2026-07-17

**Authors:** Anyan Li, Shimei Xu, Ting Li, Qiaoying Zeng, Wei Wang

**Affiliations:** 1 College of Veterinary Medicine, Gansu Agricultural University, Lanzhou 730070, China; lianyan@smorganic.cn; 2 Organic Milk Production Base, Inner Mongolia Shengmu Hi-Tech Animal Husbandry Co., Ltd., Hohhot 010111, China; 3 State Key Laboratory of Reproductive Regulation and Breeding of Grassland Livestock (Co-Established by Province and Ministry), College of Life Sciences, Inner Mongolia University, Hohhot 010070, China

**Keywords:** BoHV-1, infectious bovine rhinotracheitis, gD protein, eukaryotic expression, indirect ELISA

## Abstract

To develop an indirect ELISA kit for detecting serum antibodies against bovine herpesvirus 1 (BoHV-1), the gD protein of BoHV-1 was expressed in CHO-S cells and purified for use as the coating antigen. The optimized conditions were as follows: a coating antigen concentration of 2 µg/mL; serum dilution at 1:40 with incubation at 37 °C for 60 min; enzyme-labeled antibody dilution at 1:5000 with incubation at 37 °C for 60 min; and color development with TMB substrate at 37 °C for 15 min. The absorbance was then measured with a microplate reader. The kit could detect BoHV-1-positive serum at a dilution of up to 1:3200, showing favorable analytical detection capacity. No cross-reactivity was observed with positive sera against bovine viral diarrhea virus (BVDV), Akabane virus (AKAV), bovine parainfluenza virus type 3 (BPIV3), or lumpy skin disease virus (LSDV), reflecting ideal analytical specificity. Based on 126 field serum samples tested against a commercial IDEXX gB-based ELISA kit, we further calculated the diagnostic sensitivity (83.54%; 95% CI: 73.22–90.17%) and diagnostic specificity (100%; 95% CI: 92.43–100%) to quantitatively characterize its clinical detection performance. The ROC curve analysis based on IDEXX gB serostatus rather than true VNT-confirmed infection generated an AUC of 0.912 (95% CI: 0.856–0.951), which only reflects inter-assay concordance instead of absolute diagnostic validity. This indirect ELISA can be applied to epidemiological investigation and evaluation of immunization efficacy.

## Simple Summary

Infectious bovine rhinotracheitis (IBR) caused by bovine herpesvirus 1 severely harms cattle farming, and affordable, reliable antibody testing tools are urgently needed for large-scale farm screening. Commercial detection kits are costly, while proteins produced by common bacterial expression systems lack natural protein modifications and may weaken detection performance. This study produced the viral gD protein using mammalian CHO cells and then built a simple antibody test method. After full optimization, the test showed no false-positive signals against four common cattle viruses, could detect weakly positive serum diluted 3200 times, and had stable repeated test results. When testing 126 real farm blood samples, this method reached 100% accuracy for negative cattle and a 83.54% detection rate for infected cattle compared with imported commercial kits. This low-cost test can support regular disease surveys and vaccine effect checks on cattle farms.

## 1. Introduction

*Bovine herpesvirus type 1* (BoHV-1), the causative agent of infectious bovine rhinotracheitis (IBR), represents a significant veterinary and economic burden on the global cattle industry [1]. The virus, belonging to the genus *Varicellovirus* within the subfamily *Alphaherpesvirinae* (family *Herpesviridae*), induces a spectrum of clinical manifestations, including respiratory disease, abortion, infertility, and conjunctivitis, resulting in substantial production losses and trade restrictions [2]. Despite the implementation of eradication programs in several European countries through test-and-cull strategies and marker vaccination [3,4], BoHV-1 remains endemic in many regions worldwide [5,6], particularly in high-density livestock systems where biosecurity measures are inconsistently applied [7,8]. The biological characteristics of BoHV-1 complicate disease control efforts. Following primary infection, the virus establishes lifelong latency in sensory neurons, with periodic reactivation and viral shedding triggered by stress or immunosuppression [9,10]. This latency–reactivation cycle facilitates silent viral transmission within herds and renders complete eradication challenging [11]. Consequently, early detection of infected animals through accurate serological surveillance constitutes a cornerstone of IBR control programs [3].

Among the viral structural proteins, glycoprotein D (gD) has emerged as a critical target for diagnostic and vaccine development [12]. As an essential envelope glycoprotein, gD mediates viral attachment and entry into host cells by interacting with specific cell surface receptors [13]. Importantly, gD elicits robust humoral and cell-mediated immune responses and serves as the primary target for virus-neutralizing antibodies during natural infection [12,14]. These immunological properties, combined with its structural conservation across viral strains, render gD an ideal antigen for serodiagnostic applications and subunit vaccine design [12,15].

Currently available commercial ELISA kits for BoHV-1 serodiagnosis, such as the IDEXX IBR gB X3 antibody test and PrioCHECK™ BHV-1 gB ELISA, demonstrate high diagnostic performance validated against the virus neutralization test (VNT) but remain prohibitively expensive for large-scale application in resource-limited settings [16,17]. While previous studies have successfully employed Escherichia coli and baculovirus expression systems for gD production [14], these platforms often yield proteins with limited post-translational modifications, potentially compromising antigenicity and analytical sensitivity.

Chinese hamster ovary (CHO) cells are the predominant cellular host for the industrial production of complex therapeutic glycoproteins, accounting for over 70% of approved recombinant biopharmaceuticals [18]. These cells provide authentic mammalian post-translational modifications, including complex N-glycosylation [19], and are amenable to scalable suspension culture in chemically defined media [20]. However, the application of CHO cell-expressed gD for BoHV-1 serodiagnosis remains unexplored.

In this study, we employed CHO-S cells for eukaryotic expression of codon-optimized BoHV-1 gD protein and established an indirect ELISA method for detecting BoHV-1-specific antibodies. By optimizing critical parameters, including antigen coating concentration, serum dilution, and detection conditions, we developed a cost-effective serological assay with favorable analytical performance. Notably, most previous gD-based ELISA assays only reported analytical detection limits and cross-reactivity data without formal calculation of diagnostic sensitivity and specificity against reference commercial kits, which hinders direct comparison of clinical diagnostic capacity. To fill this gap, we supplemented quantitative calculation of clinical diagnostic indicators and ROC analysis using paired field serum samples with IDEXX gB ELISA as the reference benchmark. The validation of this method against a commercial reference kit and its application to field serum samples demonstrate its potential utility for epidemiological surveillance and immunization efficacy evaluation in endemic regions.

## 2. Materials and Methods

### 2.1. Plasmids, Cells and Serum

The recombinant eukaryotic expression vector pcDNA3.1(-) -BoHV-1-gD was synthesized by Nanjing Jinsirui Biological Technology Co., Ltd., Nanjing, China. CHO-S cells (Gibco, Thermo Fisher Scientific, Waltham, MA, USA), BoHV-1-negative and -positive serum, bovine parainfluenza virus type 3-positive serum, bovine viral diarrhea virus-positive serum, and Akabha disease virus-positive serum were stored and provided by Inner Mongolia Mongolia Microbial Technology Co., Ltd., Hohhot, China. The positive serum of bovine nodular skin disease virus was donated by Jinyu Baoling Company, Hohhot, China.

A total of 126 bovine field serum samples were randomly collected from dairy herds in Bayannur City (Inner Mongolia, China) between March and August 2025, including asymptomatic latent carriers, vaccinated cattle and unvaccinated finishing cattle of different ages. All serum samples were separated, heat-inactivated and preserved at −20 °C prior to testing.

### 2.2. Main Reagents

Ampicillin sodium and the SDS-PAGE gel kit were purchased from Beijing Solaibao Technology Co., Ltd., Beijing, China. The endotoxin-free plasmid extraction kit was purchased from Nanjing Nuoweizan Company, Nanjing, China; the transfection reagent was purchased from Sipeng Biotechnology Co., Ltd., Suzhou, China; M5 HiPer plus Taq HiFi PCR mix (with blue dye), agarose for M5 DNA electrophoresis, M5 HiPure Gelred Plus nucleic acid dye, and M5 Prestained Protain Ladder (10–180 kda) were purchased from Beijing Jumei Biotechnology Co., Ltd., Beijing, China; DL 1000 DNA Marker was purchased from TaKaRa, Kyoto, Japan; and the BeyoGoldTM His label purification kit was purchased from Shanghai Biyuntian Biotechnology Co., Ltd., Shanghai, China. Horseradish peroxidase-conjugated rabbit anti-bovine IgG and fish gelatin were purchased from Sigma, St. Louis, MO, USA; Hrp-labeled murine IgG, goat anti-mouse IgG and SuperKineTM ECL solution were purchased from Yacoin Biotechnology Co., Ltd. Shanghai, China; Bovine serum albumin was purchased from Beijing Coolaibo Technology Co., Ltd., Beijing, China.

### 2.3. Test Methods

#### 2.3.1. Construction of Plasmids

According to the sequence of the BoHV-1 gD gene in the NCBI database (GenBank accession number: JN787954.1), the target gene was codon-optimized to match CHO cell expression preference by Jinrui Biotechnology Co., Ltd., Chengu, China and the optimized gene was synthesized into the pcDNA3.1(-) vector.

#### 2.3.2. Expression and Purification of Recombinant gD Protein

The recombinant plasmid pcDNA3.1(-) -BoHV-1-gD was transfected into CHO-S cells and incubated at 37 °C with shaking at 125 r/min. The expression of BoHV-1-gD was verified by SDS-PAGE and Western blot. The protein immunoreactivity was verified by Western blot and purified via Ni-affinity chromatography using a His-tag purification kit (Thermo Fisher Scientific Inc, Waltham, MA, USA). BoHV-1-positive serum was used as the primary antibody (1:1000 dilution), and HRP-conjugated rabbit anti-bovine IgG as the secondary antibody (1:10,000 dilution).

The transfected cell suspension was disrupted by sonication (150 W; 2 s on/2 s off cycle). The lysate was collected, and the pellet was resuspended in PBS and loaded onto an equilibrated Ni column for protein purification. The purified protein was identified by SDS-PAGE.

#### 2.3.3. Establishment of an Indirect ELISA Method

The purified gD antigen was serially diluted to 4.000, 2.000, 1.000, and 0.500 µg/mL for checkerboard coating. BoHV-1-positive and -negative sera were diluted at 1:10–1:80 to screen for the optimal serum dilution. HRP rabbit anti-bovine IgG served as the secondary antibody; blocking buffers, including 5% skim milk, 1% skim milk, 5% BSA, 1% BSA, 3% fish gelatin, and 0.5% fish gelatin, were compared via the P/N ratio to select the optimal blocking condition. After primary and secondary antibody optimization, TMB single-component substrate was incubated for 5, 10, 15, 20, and 25 min to confirm optimal color development time.

#### 2.3.4. Critical Value Determination

A total of 74 confirmed BoHV-1-negative sera were tested under optimized conditions to calculate the mean and standard deviation of the S/P ratios. The cut-off value was defined as mean S/P + 3SD; samples with S/P ≥ 0.1686 were judged as positive and those with S/P < 0.1686 as negative.

#### 2.3.5. Analytical Cross-Reactivity Test (Analytical Specificity Evaluation)

Cross-reaction assays were conducted using BVDV-, AKAV-, BPIV3- and LSDV-positive sera, with the OD450 and S/P values recorded to evaluate the analytical specificity of the gD ELISA.

#### 2.3.6. Analytical Detection Limit Test (Analytical Sensitivity Evaluation)

BoHV-1-positive serum was serially diluted starting at 1:100 to detect the maximum dilution with a positive signal, which represented the analytical detection capacity of the assay.

#### 2.3.7. Indirect ELISA Reproducibility Test

Four serum samples were tested in triplicate within the same plate for intra-batch repeatability; three batches of purified gD coating antigen were used for inter-batch testing. The mean OD values and coefficient of variation (CV) were calculated for evaluation.

#### 2.3.8. Indirect ELISA Concordance Rate Test

The homemade gD ELISA and commercial IDEXX IBR gB X3 ELISA were run in parallel on all 126 field sera. A 2 × 2 contingency table was built to calculate concordance, diagnostic sensitivity (DSn) and diagnostic specificity (DSp):

Diagnostic Sensitivity = TP/(TP + FN) × 100%

Diagnostic Specificity = TN/(TN + FP) × 100%

TP = double-positive samples; FN = IDEXX-positive, homemade ELISA-negative; TN = double-negative; and FP = IDEXX-negative, homemade ELISA-positive.

ROC curve analysis was constructed based on the continuous S/P values of all 126 samples, with IDEXX gB serostatus as the reference benchmark rather than true infection validated by VNT.

## 3. Results

### 3.1. Expression and Purification of the gD Protein

**Figure 1:**
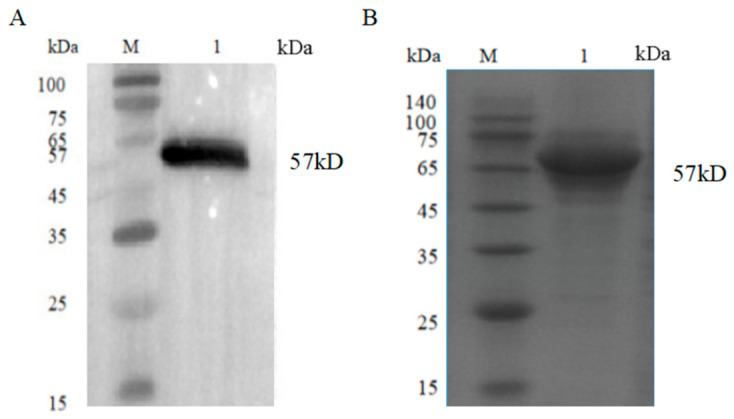
Western blot and SDS-PAGE identification of recombinant BoHV-1 gD protein expressed in CHO-S cells. M: Pre-stained protein molecular weight marker; (**A**) Western blot of transfected cell culture supernatant (primary antibody: BoHV-1-positive bovine serum (1:1000); secondary antibody: HRP-conjugated rabbit anti-bovine IgG (1:10,000)). The slight smear of the ~57 kDa band in Panel A was attributed to mild protein degradation during supernatant collection and excessive protein loading volume. (**B**) SDS-PAGE analysis of nickel-column-purified gD protein with distinct single target band at 57 kDa. A1 = cell culture supernatant after transfection; B1 = purified gD protein.

Western blot using BoHV-1-positive primary antibody detected a target band at ~57 kDa in the cell culture supernatant (Figure 1A), confirming the specific immunoreactivity of recombinant gD. After Ni-column purification, SDS-PAGE displayed a single dominant band at the expected molecular weight of 57 kDa (Figure 1B).

### 3.2. Establishment of the Indirect ELISA Method

Checkerboard titration and optimization of all reaction parameters yielded the optimal ELISA system shown in Table 1.

### 3.3. Determination of the Critical Value for the Indirect ELISA Method

The average S/P value of 74 negative sera was 0.0150 with SD = 0.0512, giving a cut-off S/P = 0.1686.

ROC curve analysis was performed using IDEXX gB serological results as the reference standard, not the true BoHV-1 infection status confirmed by VNT. The AUC value reached 0.912 (95% CI: 0.856–0.951), which only reflects the concordance between two ELISA platforms instead of absolute independent diagnostic performance. This cut-off value exhibited reliable discrimination between antibody-positive and -negative samples under the IDEXX-matched comparison system.

### 3.4. Analytical Cross-Reactivity (Analytical Specificity) Results

No elevated OD/S/P signals were observed for heterologous viral-positive sera (Table 2), proving ideal analytical specificity without cross-reaction.

### 3.5. Analytical Detection Limit (Analytical Sensitivity) Results

Positive serum diluted up to 1:3200 still generated an S/P above the cut-off value (Table 3), demonstrating excellent analytical detection capacity.

### 3.6. Repeatability Experiment Results

Intra-batch maximum CV = 8.8%; inter-batch maximum CV = 8.85%. All CV values were below the 10% threshold, indicating stable repeatability (Table 4).

### 3.7. The Result of the Compliance Rate Test

Parallel testing of 126 field sera yielded the 2 × 2 contingency data shown in Table 5: TP = 66, FN = 13, TN = 47, and FP = 0.

Calculated diagnostic sensitivity = 83.54% (95% binomial CI: 73.22–90.17%); diagnostic specificity = 100% (95% binomial CI: 92.43–100%). All 13 discordant samples were IDEXX gB-positive but gD-ELISA-negative, defined as false negatives under this comparison system. The overall sample concordance rate reached 89.68%.

## 4. Discussion

In recent years, with the rapid growth of the global cattle population and increased frequency of cattle movement between regions, the spread and prevalence of IBR have further intensified, presenting new challenges for prevention and control efforts [21]. BoHV-1 is a highly contagious virus with latent infection characteristics. After infection, it can cause upper respiratory tract diseases, conjunctivitis, reproductive tract diseases, abortion, and immunosuppression in cattle, leading to secondary infections and causing significant economic losses [22]. Following acute infection, BoHV-1 can establish lifelong latency in sensory neurons [9]. Induced by stress or hormonal fluctuations, it can undergo periodic reactivation and viral excretion, enabling hidden transmission within the cattle herd, making the complete eradication of this virus extremely challenging [23].

Currently, vaccination is the primary means of controlling this disease; however, existing vaccines cannot prevent the establishment of latent BoHV-1 infection [24]. Furthermore, the virus is highly adaptable to its host, and even vaccination cannot completely prevent infection [24]. Consequently, it will take a long time for control programs to eradicate the disease. Against this backdrop, early, accurate, and rapid diagnosis is crucial for implementing effective prevention and control measures. Current serological methods for BoHV-1 antibody detection primarily include the virus neutralization test (VNT) and ELISA. VNT is widely regarded as the gold standard for BoHV-1 antibody detection; however, it involves the use of live viruses and requires complex cell culture facilities, making it unsuitable for large-scale sample testing [5]. In contrast, the ELISA method has been widely adopted in serological surveillance and eradication programs for IBR due to its stable analytical performance, low testing cost, good reproducibility, and ease of standardization [3].

In terms of antigen selection for diagnosis, the gD glycoprotein has been identified as a key target for BoHV-1 diagnosis and vaccine development [25]. gD is a major molecular component on the surface of viral particles and in infected cells, playing a central role in viral adsorption and invasion of host cells [24], while also inducing high levels of neutralizing antibodies and cell-mediated immune responses [25]. Due to the high structural conservation of gD across different BoHV-1 strains (97–100% homology among 24 global strains), gD-based ELISA assays hold great promise for epidemiological surveillance [25]. Nautiyal et al. (2023) established an indirect ELISA using *E. coli*-expressed gD, with diagnostic sensitivity of 82.9% and specificity of 91.3%, validated by the VNT gold standard [5].

However, prokaryotic and baculovirus expression systems produce gD with incomplete post-translational modifications, which may impair antigen binding and lower analytical detection capacity [25]. CHO mammalian cells can generate recombinant glycoproteins with native N-glycosylation, disulfide bonds and tertiary folding [22,26,27,28,29]. Therefore, this study expressed codon-optimized BoHV-1 gD in CHO-S cells and constructed a low-cost indirect ELISA, which outperforms prokaryotic gD ELISA in theoretical antigenicity due to complete glycosylation modification [19].

The developed ELISA displayed reliable analytical performance: no heterologous cross-reaction with four common bovine pathogens, a detection limit of 1:3200 for positive serum, and intra/inter-batch CV < 10, showing stable repeatability. Parallel testing against commercial IDEXX gB ELISA achieved 89.68% sample concordance.

It should be noted that the IDEXX kit targets gB antibody, while our assay detects the gD antibody; gB elicits earlier, longer-lasting humoral responses during BoHV-1 infection, whereas latent cattle under low viral reactivation often produce low gD antibody titers. This immunological difference likely contributes to the 13 discordant serum samples observed in this study. Without paired antibody titer and latent infection data from these 13 animals, we cannot confirm it as the definitive sole cause, and the weaker general analytical sensitivity of gD-based ELISA also represents a plausible explanation for the mismatch results.

Notably, the IDEXX gB ELISA has certified diagnostic sensitivity (97.4%) and specificity (99.8%) against the VNT gold standard [29], far higher than the 83.54% diagnostic sensitivity of our gD ELISA relative to the gB platform. The moderate diagnostic sensitivity of our assay brings an inherent risk of false-negative detection results during field screening. Critically, this false-negative risk becomes substantially more prominent in cattle herds with low BoHV-1 seroprevalence, where latent carriers are easily missed in epidemiological surveys. This limitation should be fully considered when deploying this homemade ELISA for large-scale farm monitoring.

Although commercial kits deliver superior diagnostic accuracy, their high procurement cost restricts wide use in low-resource breeding regions. Our assay achieves 100% diagnostic specificity (95% CI: 92.43–100%) and acceptable analytical performance while cutting per-sample testing expense, offering an economical alternative for routine serological surveillance and vaccine efficacy evaluation.

Several objective limitations of this research require acknowledgement.

First, we only conducted comparative testing with a gB-based commercial kit rather than the recognized VNT serological gold standard, so AUC and diagnostic metrics merely reflect inter-kit concordance instead of absolute true diagnostic capacity against actual infection status.

Second, although CHO expression theoretically supports full gD glycosylation, we did not perform glycoproteomic sequencing to directly verify the glycan profile of the recombinant protein, which lacks direct experimental evidence for its structural advantage.

Third, the cross-reactivity pathogen panel only covers four viruses. Key co-infectious agents, including bovine respiratory syncytial virus (BRSV) and bovine coronavirus (BCoV), were not included, and we plan to complete this expanded cross-reactivity verification in subsequent research.

In summary, this study successfully expressed immunocompetent BoHV-1 gD protein via the CHO-S eukaryotic system and established a novel indirect ELISA for BoHV-1 antibody detection. The assay possesses ideal analytical specificity, high analytical detection capacity and stable repeatability, with acceptable concordance with commercial kits. Nevertheless, its relatively low diagnostic sensitivity creates false-negative risks, especially in low-prevalence herds, which must be taken into account in field application. With low cost and simple operation, this method can serve as a supplementary screening tool for IBR epidemiological monitoring and post-vaccination efficacy assessment in Chinese cattle herds.

## 5. Conclusions

This study successfully expressed the BoHV-1 gD protein in CHO-S cells and established an indirect ELISA using purified gD as the coating antigen. After systematic parameter optimization, the optimal reaction system was confirmed: 2 µg/mL of the coating antigen, serum dilution at 1:40, secondary antibody at 1:5000, 5% skim milk blocking, and TMB incubation for 15 min at 37 °C.

The ELISA exhibits favorable analytical detection capacity with a positive serum limit of 1:3200 and no cross-reactivity against four heterologous bovine viral sera. Quantitative comparison with IDEXX gB ELISA yielded a diagnostic sensitivity of 83.54% (95% CI: 73.22–90.17%) and a diagnostic specificity of 100% (95% CI: 92.43%). The intra- and inter-batch coefficients of variation were both less than 10%, demonstrating stable repeatability. A total of 126 clinical serum samples reached an 89.68% concordance rate with the commercial kit. In conclusion, this low-cost, easy-to-operate indirect ELISA is suitable for routine serological surveillance and immunization efficacy evaluation of IBR, with awareness of its moderate diagnostic sensitivity and associated false-negative risks in low-prevalence herds.

## Figures and Tables

**Table 1:** ELISA reaction conditions.

	Coating Concentration	Blocking Conditions	Serum Dilution Ratio	Enzyme-Labeled Antibody Dilution Ratio	Substrate
Optimal mass concentration	2 μg/mL	5% skim milk powder	1:40	1:5000	
Reaction conditions	4 °C, 14 h	37 °C, 1 h	37 °C, 1 h	37 °C, 1 h	37 °C, 15 min

**Table 2:** Analytical cross-reactivity test results (analytical specificity).

	Bovine Viral Diarrhea Virus (BVDV)	Akabane Virus (AKAV)	Bovine Parainfluenza Virus Type 3 (BPIV3)	Bovine Nodular Dermatosis Virus (LSDV)	Positive	Negative
OD_450nm_	0.2753	0.1501	0.2439	0.3762	1.5535	0.1472
S/P	0.0911	0.0021	0.0688	0.1628		

**Table 3:** Analytical detection limit test results (analytical sensitivity).

	1:100	1:200	1:400	1:800	1:1600	1:3200	1:6400	Positive	Negative
OD_450nm_	1.8761	1.6119	1.3433	1.0825	0.8031	0.4552	0.1383	1.6265	0.1532
S/P	1.1694	0.9901	0.8078	0.6308	0.4411	0.2050	−0.1011		

**Table 4:** Repeatability experiment results.

Sample	Intra-Batch Repeatability Test				Inter-Batch Repeatability Test			
	Replicate Wells (OD Value)			Coefficient of Variation (CV) (%)	Replicate Wells (OD Value)			Coefficient of Variation (CV) (%)
1	1.3615	1.3774	1.347	1.12	3.0974	3.1952	2.9283	4.39
2	1.9533	2.2472	1.9671	8.07	3.1144	3.0152	3.1095	1.82
3	3.6542	3.7433	3.3752	5.35	0.0953	0.0919	0.1002	4.36
4	1.8451	1.9733	1.9015	3.37	2.3193	2.1933	2.2297	2.89

**Table 5:** The result of the coincidence rate experiment.

Indirect ELISA Detection Method	IDEXX gB X3 ELISA		Total
	+	−	
+	66	0	66
−	13	47	60
Total	79	47	126
Compliance rate	89.68%		

## Data Availability

The original contributions presented in this study are included in this article. Further inquiries can be directed to the corresponding authors.
